# Analysis of alcoholic EEG signals based on horizontal visibility graph entropy

**DOI:** 10.1007/s40708-014-0003-x

**Published:** 2014-09-13

**Authors:** Guohun Zhu, Yan Li, Peng (Paul) Wen, Shuaifang Wang

**Affiliations:** University of Southern Queensland, West Street, Toowoomba, QLD 4350 Australia

**Keywords:** Multi-channel EEG, Alcoholism, Graph entropy, Slow waves, Classification

## Abstract

This paper proposes a novel horizontal visibility graph entropy (HVGE) approach to evaluate EEG signals from alcoholic subjects and controlled drinkers and compare with a sample entropy (SaE) method. Firstly, HVGEs and SaEs are extracted from 1,200 recordings of biomedical signals, respectively. A statistical analysis method is employed to choose the optimal channels to identify the abnormalities in alcoholics. Five group channels are selected and forwarded to a K-Nearest Neighbour (K-NN) and a support vector machine (SVM) to conduct classification, respectively. The experimental results show that the HVGEs associated with left hemisphere, $C$1, $C$3 and *FC*5 electrodes, of alcoholics are significantly abnormal. The accuracy of classification with 10-fold cross-validation is 87.5 $\%$ with about three HVGE features. By using just optimal 13-dimension HVGE features, the accuracy is 95.8 $\%$. In contrast, SaE features associated cannot identify the left hemisphere disorder for alcoholism and the maximum classification ratio based on SaE is just 95.2 $\%$ even using all channel signals. These results demonstrate that the HVGE method is a promising approach for alcoholism identification by EEG signals.

**Electronic supplementary material:**

The online version of this article (doi:10.1007/s40708-014-0003-x) contains supplementary material, which is available to authorized users.

## Introduction

Alcoholism is a common neurological disorder caused by the mutual effect of genetic and environmental factors. It not only damages the brain system but also leads to cognitive and mobility impairments [1]. These impairments may lead to serious accidents while driving and operating machineries [2]. The World Health Organization (WHO) [3] reported that alcohol abuse is the third highest risk factor for causing diseases and results in 2.5 million deaths each year. How to distinguish alcoholics from normal subjects in a reliable way will not only reduce unnecessary economic losses and social problems, but also provide a quick and easy way for doctors in the clinical settings. Electroencephalogram (EEG) is a very effective tool for studying the complex dynamics of brain activities. It can visualize complex brain activities as dynamic outputs. Therefore, it can be used to distinguish alcoholics from normal subjects based on the differences in the signals, which aids in the detection and diagnosis of alcoholics.

Frequency-domain analysis and time-domain methods are widely used to assess alcoholic EEG or EOG signals. Hayden et al. [4] claimed by analysing the frequency power that alcoholism in people may lead to frontal lobe dysfunction. Michael et al. [5] found the alpha-wave coherence increases at the central region and the appearance of a slow-beta coherence. Winterer et al. [6] reported that the alcoholic subjects showed increased left-temporal alpha coherence and slow-beta coherence. Hughes et al. [7] noticed that both EEG and QEEG reveal marked abnormalities in alcoholic subjects, which might result in increased slow activity or the converse, while Waite et al. [8] using QEEG techniques confirmed that slow waves dominated the brain of alcoholics. By using an event-related potential technique, Zhang et al. [9] suggested the right hemisphere dysfunction in alcoholics unlike that of drinking controlled subjects. Zhu et al. [10] studied the alcoholism using a likelihood synchronization method and found that there is a slight difference between alcoholics and normal persons based on EOG signals. However, most of these reports did not match the recent finding based on the study of brain tissue mitochondria [11] or fMRI detection [12] that alcohol significantly reduced amygdala reactivity to threat signals. New nonlinear methods are needed to investigate the alcoholic EEG or EOG signals.

Currently, some nonlinear EEG complexity analysis methods, such as correlation entropy [2], sample entropy [13] and Omeg-complexity [14], have been applied to analyse the alcoholic EEG signals. In general, the input signals are more random, and the value of entropy is large. Based on this principle, Zhou et al. [13] showed that the EEG signals of alcoholics are more random than those of normal using a sample entropy (SaE) method. In contrast, SaE of heavy drinkers is less than healthy during driving [2], while correlation entropy is reverse. Thus, biologists are finding these contradictions very difficult to interpret. Thus, it is necessary to find a good entropy to measure the complexity of the alcoholics and the results can be interpreted from the biological point of view.

This paper uses HVGE features to analyse the alcoholic EEG signals from multi-channel EEG signals. Although the concept of HVGE has been appearing for a long time [15], HVGE has been only recently applied to social networks [16] and data mining [17]. The HVGE has never been applied to analyse the alcoholic EEG signal processing as far as we know. To show the outcome, the SaEs are compared to study at the same three steps. At first, the HVGE and the SaE features are extracted from multi-channel EEG and EOG signals to distinguish alcoholics from controlled drinkers. Each of EEG and EOG recordings in 1 s from 63 channels is mapped to 63 HVG. There are 1,200 EEG recordings to be analysed. Totally, 76,800 HVGE and SaE features are extracted. Then all the HVGE features are evaluated with Kruskal-Wallis test to identify the abnormal channels. Finally, the features from one channel, three left hemisphere channels, 13 optimal channels and 63 channels are forwarded to a K-Nearest Neighbour (K-NN) and a support vector (SVM) to conduct classification, respectively.

This paper is organized as follows: the experimental data and the proposed graph entropy and other related methods are introduced in Sect. 2. Section 3 presents experimental data and the experimental results. Finally, conclusions are drawn in Sect. 5.

## The methods

### Horizontal visibility graphs

**Fig. 1:**
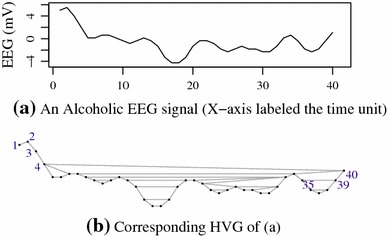
An alcoholic EEG (**a**) and the corresponding HVG (**b**)

A horizontal visibility graph (HVG) is a kind of complex networks [18]. Normally, a time series $\{x_i\}_{(x=i,\ldots ,n)}$ is mapped into a graph $G(V, E)$, where a time point $x_i$ is mapped into a node $v_i \in V$. The relationship between any two points $\left( x_i, x_j\right)$ is represented by an edge $e_{ij}$, which are connected if and if only the maximal values between $x_i$ and $x_j$ are less than both of them. Our previous work [19] shows that the edge can be defined as

1 $$ w_{ij} = \left\{\begin{array}{ll} &1, x_j>{\mathrm{max}}\left( x[(j+1) \ldots (i-1)]\right) \\ &1, j+1=i \\ &0, {\mathrm{otherwise}}. \end{array} \right. $$

where $e_{ij}=0$ implies that the edge does not exist, otherwise it does. Figure 1 shows an HVG associated with an alcoholic EEG time series, which was collected from electrode $FP1$ of subject $co2a0000368$ [20] in alcoholic EEG datasets.

The number of time points in Fig. 1a is 40. The first node in Fig. 1b is associated with the first point in Fig. 1a. The second node corresponds to the second point of the time series and so on. In a complex network, the node degree and degree sequence are the two basic characteristics of graph. A degree $d(v_i)$ of node $v_i$ is the number of connected edges from $v_i,$ while a degree sequence (DS) is the sequence of the degree of a graph.

#### *Example 1*

: Let $Y$ denote the first 12 values shown in Fig. 1a (5.015, 5.503, 4.039, 2.085, 0.132, 0.132, 0.621, 0.621, 0.132, $-$0.356, $-$0.844, $-$0.356) and in Fig. 1b, $d_1=1$ and $d_2=2$. The DS of the HVG associated with $Y$ is (1, 2, 2, 3, 2, 2, 3, 2, 2,3 ,2, 2).

### Graph entropy

There are several graph entropy calculation methods based on either vertex or edges [15]. This study defines the graph entropy (GE) with Shannon’s entropy formula [21] measurement as shown:

2 $$ h=-\sum _{i=1}^{n}{p(k)\log (p(k))},$$

where $p(k)$ is the degree distribution of the graph $G$.

The degree distribution (DD) is a probability degree $k$ over a DS. It is obtained by counting the number of nodes having degree k divided by the size of the DS. Following example 1, the $p(k)$ of the DS in that example is (0, 1/12, 8/12, 3/12). Then the graph entropy is $0.824$ when the logarithm operator is based on two. It is obvious that the more fluctuating the degree sequence is, the larger the graph entropy is. In other words, the more regular a graph is, the smaller its graph entropy is.

### Sample entropy

Sample entropy (SE) was proposed by Richman and Moorman [22]. It has been used to measure the complexity of alcoholic EEG [13], epileptic EEG [23, 19] and other EEG signal processing. An SaE algorithm used in this study to estimate the SaE is available in the Physione website (http://www.physionet.org/physiotools/sampen/). The algorithm of SampEn has three input parameters: (1) $$m$$: the embedded dimension, (2) $$r$$: the similarity criterion, (3) $$n$$: the length of a time series $$\{ x_i \}_{i=1,2,\ldots ,n}$$. In this study, two SaE features ($$Se_{1}$$: $$m=2$$, $$r=0.15$$$$Se_{2}$$: $$ m=2$$, $$ r=0.2$$) of each epoch of EEG signals are extracted.

### K-Nearest Neighbour (K-NN) algorithm

To compare the performances of HVGE based on HVGs, a K-Nearest Neighbour (K-NN) algorithm is selected to conduct the binary classification. K-NN algorithm is a traditional pattern recognition method, which is a statistical supervised classification. The idea is that given a new test data *t*, the algorithm obtains the $K-$ nearest neighbours from the training set based on the distance between t and the training set. The most dominated class amongst these K neighbours is assigned as the class of $t$. In this study, the K-NN algorithm is included in R package (FNN; http://cran.r-project.org/web/packages/FNN/index.html), where K is assigned as $3$ without considering the optimal case.

### SVM

To measure the performance of the HVGE features from 63 electrodes, a support vector machine (SVM) is selected to conduct the binary classification. The SVM has been successfully used to classify the HVG features associated with sleep EEG signals [24]. It can perform both the linear space discrimination and nonlinear classification by choosing different *kernel* functions which can be linear, polynomial kernel, radical basis function (RBF) and sigmoid. In this paper, the SVM algorithm with RBF kernel is implemented in R package $e1071$ [25]. The RBF kernel was applied. When optimal case is not considered, two parameters, cost $C$ and $\sigma$ , of the RBF kernel of SVM are fixed to 10 and 0.1, respectively.

## Experimental data and results

The experiments include two parts: (1) analysing alcoholic EEGs based on the HVGE and SaE of the HVGs and (2) evaluating the classification accuracy by HVGE and SaE separately. All algorithms are conducted by C language and R language. All experiments are implemented on a computer with 3.0 GHz Inter CoreTM Duo E8400 processor and 4 GB of RAM.

### Experimental data

The experimental data used in this paper were obtained from the University of California, Irvine Knowledge Discovery in Databases Archive UCI KDD [20]. They were collected from 122 subjects. Each subject completed 120 trials with three types of stimuli [9]. The recordings from a subject include $61$ channel EEG signals, two EOG channels and one reference electrode. The sampling rate of all channel data is 256 Hz, and the duration of each trial is one second. There are three datasets which are **SMNI_CMI_TRAIN**, **SMNI_CMI_TEST** and **FULL**, respectively. In this study, only the first two databases are used because **FULL** datasets contain a few all-zero recordings [10]. There are 600 recorded files in **SMNI_CMI_TRAIN**, with each recording containing the signals from 64 electrodes caps. The indices of the 64 electrodes are “*FP1*”, “*FP2*”, “*F7*”, “*F8*”, “*AF1*”, “*AF2*”, “*FZ*”, “*F4*”, “*F3*”, “*FC6*”, “*FC5*”, “*FC2*”, “*FC1*”, “*T8*”, “*T7*”, “*CZ*”, “*C3*”, “*C4*”, “*CP5*”, “*CP6*”, “*CP1*”, “*CP2*”, “*P3*”, “*P4*”, “*PZ*”, “*P8*”, “*P7*”, “*PO2*”, “*PO1*”, “*O2*”, “*O1*”, “*X*”, “*AF7*”, “*AF8*”, “*F5*”, “*F6*”, “*FT7*”, “*FT8*”, “*FPZ*”, “*FC4*”, “*FC3*”, “*C6*”, “*C5*”, “*F2*”, “*F1*”, “*TP8*”, “*TP7*”, “*AFZ*”, “*CP3*”, “*CP4*”, “*P5*”, “*P6*”, “*C1*”, “*C2*”, “*PO7*”, “*PO8*”, “*FCZ*”, “*POZ*”, “*OZ*”, “*P2*”, “*P1*”, “*CPZ*”, “*nd*” and “*Y*”. The electrodes X and Y are EOG signals, and $nd$ are reference electrodes. The $nd$ are removed in our analysis. Thus, features are extracted from 63 channels.

### Comparisons of HVGE and SaE indices between alcoholics and drinking controlled subjects

Average HVGE and SaE of 63-channel EEG signals are drawn in supplementary Fig. S1. Compared the average HVGE of alcoholic with those of controlled drinkers, the results show that the HVGE associated with alcoholic EEG signals are lower than those of controls in $42/63$ channels. In contrast, the SaE features are higher than those of controls except for two channels: $C1$ and $C2$. In order to observe clearly, 13 optimal HVGE and SaE channels are selected by statistical test.

**Fig. 2:**
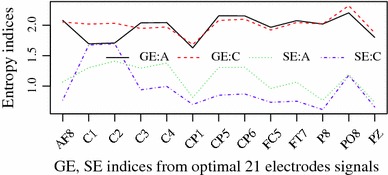
X-axis is indices of 13 optimal channels and Y-axis the mean HVGE and SaE (SaE value times two for clear comparison) (*A* alcoholic, *C* controlled drinkers)

The optimal channels are selected by the following two tests: Firstly, Shapiro-Wilk tests show that the HVGE of both EEG and EOG signals does not satisfy normal distributions. Then non-parametric Wilcoxon tests are applied to select the HVGEs from different channels. According to the statistical significance ($p<0.001$), 13 optimal channels are selected as shown in Fig. 2. In contrast to alcoholic's SaE decrease, the HVGE of $C1$ and $C2$ from the same group are significant increase. SaEs are always higher in alcoholic, whereas only seven HVGEs of alcoholic, $AF$8, $C$3, $C$4, *CP*5, *CP*6, *FC*5 and *TP*7, are larger than those of controlled drinkers in Fig. 2.

To show the test detail of the HVGEs between alcoholics and controlled subjects, five groups’ statistical with Wilcoxon test are listed in Table 1. The first group is channel $C1$ as it is abnormal in both HVGE and SaE. The second one is three left electrodes $C$1, $C$3 and *FC*5, because left hemisphere has been reported to be abnormal in [9]. The third group is the two EOGs as our previous results [10] showed that these two channels were not significantly different with synchronization likelihood methods. The fourth one is the selected optimal channels based on Fig. 2. The last is all 61 EEG channels.

**Table 1:** Statistical HVGE features from five group channels

Electrodes	Alcoholic	Control	$p$ value
$C$1	1.683 $\pm$ 0.465	1.984 $\pm$ 0.430	$<$0.01
$C$1, $C$3, *FC*5	1.935 $\pm$ 0.295	1.928 $\pm$ 0.310	$<$0.001
Two EOGs	1.997 $\pm$ 0.128	2.013 $\pm$ 0.235	0.07
13 EEGs	1.964 $\pm$ 0.229	1.959 $\pm$ 0.130	$<$0.001
61 EEGs	1.904 $\pm$ 0.170	1.927 $\pm$ 0.137	$<$0.001

It is clear that the HVGE of two-channel EOGs is slightly different from the alcoholics and control drinkers ($p>0.05$). However, most HVGE features of EEG signals are significant difference between these two groups.

### Comparing classification accuracy of HVGE with SaE features

**Fig. 3:**
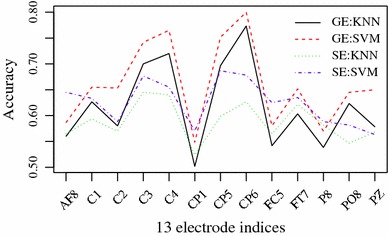
Classification for 13 channels. X-axis is electrode indices and Y-axis the accuracies using HVGE and SaE

This section investigates the HVGE and SaE features of EEG signals for identifying alcoholic from healthy subjects. The database **SMNI_CMI_TRAIN** is the training set, and the database **SMNI_CMI_TEST** is the testing set. At first, the classification accuracy of HVGE and SaE from 61-channel EEG data and two EOG signals is investigated as shown in supplementary Fig. S2. All individual channel features are forwarded to a K-NN and a SVM to conduct single-channel classification, respectively. To display clearly, the classifying results based on 13 selected channels from Fig. 2 are shown in Fig. 3. It is clear that the accuracy of SVM-based HVGE and SaE is highest at channel 20 (*CP*6). However, accuracy based on HVGE is larger than that of SaE.

Then, four group channels are illustrated in detail by comparing with the HVGE and SaE. The first group is the channel 20 (*CP*6) based on Fig. 3. The second one is the combination of three channels on the left hemisphere $C$1, $C$3 and *FC*5 based on Fig. 3 and Table 1. The third class is the 13 optimal channels selected based on Figs. 2 and 3. The last group uses all electrodes. Finally, the HVGE and SaE from these four groups of combined channels are forwarded into classifiers K-NN and SVM to conduct the classification, separately. The results are shown in Table 2.

**Table 2:** Classification accuracies of K-NN and SVM based on HVGE and SE features with four group channels

Electrodes	HVGE		SaE	
	K-NN (%)	SVM (%)	K-NN (%)	SVM (%)
*CP*6	77.2	79.1	62.7	67.8
$C$1, $C$3, *FC*5	86.5	85.5	69.5	69.5
13 channels	94.5	96.2	82.7	82.0
63 channels	97.2	96.5	92.8	85.5

It should be noticed that the performance of K-NN is better than that of the SVM on both HVGE and SaE features as shown in Table 2. Ten-fold cross-validation and grid search method [26] are applied to verify these results by elaborately selecting the kernel parameters of SVM. The K-NN method is selected as the optimal $k$ neighbours. The datasets are combined from the database **SMNI_CMI_TRAIN** and **SMNI_CMI_TEST**. The results are listed in Table 3. The performances of HVGE based on classifiers K-NN or SVM are approximate or equivalent.

**Table 3:** Ten-fold cross-validation accuracies of K-NN and SVM based on HVGE and SE features with optimal parameters

Electrodes	HVGE					SaE				
	K-NN		SVM			K-NN		SVM		
	k	Acc (%)	C	$\sigma$	Acc (%)	k	Acc (%)	C	$\sigma$	Acc (%)
*CP*6	24	79.3	8	33.47	79.4	26	75.3	0.3	23.08	76.6
$C$1, $C$3, *FC*5	9	87.5	8	0.93	87.6	23	84.5	8	0.55	83.8
$13$ channels	9	95.6	8	0.09	95.8	21	89.7	4	0.07	90.2
$63$ channels	7	98.2	4	0.01	98.1	7	95.2	8	0.01	94.3

Based on Fig. 3 and Tables 2 and 3, the proposed method based on HVGE features is better than that of SaE. As high as 98.2 % classification accuracy could be achieved when using all electrodes by K-NN. However, accuracy of 95.8 $\%$ based on 13 optimal channels is close to the highest performance. Even with three left optimal channels, the accuracy achieved is 87.6 $\%$ with a SVM classifier. Therefore, the feasibility of the proposed method in discriminating alcoholics and controlled subjects is obvious.

## Discussion

The present study demonstrates a novel HVGE to assess alcoholic EEG signals from multi-channel EEG signals. In contrast to higher sample entropy features in alcoholic EEGs, lower HVGE features dominate the alcoholic EEG and EOG signals.

The HVGE of three left hemisphere electrodes from alcoholics, $C$1, $C$3 and *FC*5, are found to be significantly abnormal, which can achieve 87.6 $\%$ of accuracy. When 13 HVGE features are applied, the classification accuracy is close to the whole 63-channel performance.

From physical point of view, higher entropies imply random signals. SaE of alcoholics is higher than those of healthy subjects as shown in Fig. 2; similarly, the alcoholic EEG signals can be explained as more random than those of healthy subjects [13]. However, high HVGE of a time series does not imply random signals. In contrast, low HVGE can be interpreted that the signals is dominated by slow frequency bands. As the presented HVGE is defined based on degree as shown in Eq. 2, a low HVGE of a graph leads to regular graph. According to Nunez et al.’s [27] results, a low period of signals leads a low mean degree of HVG. In that case, the low HVGE of alcoholics EEG is interpreted as slow frequency domination, which is different to those low SaE of the healthy EEG implied regular signals.

From biological point of view, two meaningful results of alcoholism are presented by HVGE features. The first is that the left hemisphere of alcoholic patients are significantly impacted. From Fig. 2 and Tables 1 and 2, the left hemisphere channels,$C$1, $C$3, *FC*5, are significantly different from those of healthy subjects. These results agree with the Zhang et al.’s [9] results that the left hemisphere of alcoholics are significantly impacted. The other one is that the alcoholic brains are dominated by slow waves according to the lower HVGE as shown in Table 1. According to the low HVGE of alcoholics as shown in Fig. 2 and Table 1, our results indicate that the alcoholic EEG signals possess low frequency domain, which is consistence with results of [8].

## Conclusion

This study contributed to three following facts:

First, the low HVGE of alcoholics proves that the alcoholic EEG signals are dominated by slow waves, which shows that the horizontal visibility graph of alcoholic EEG signals is more regular than those of the controlled subjects. Next, the 13 optimal channels for identifying alcoholics are selected by HVGE. Based on 10-fold cross-validation, these optimal channels can achieve 95.8 $\%$ of accuracy, and it is just 2.2 $\%$ less than the highest performance which needs 63 channels. Last, this research is the first to apply the graph entropy features to study alcoholic EEG signals. And it is also the first time several existence alcohol studies are confirmed from a public alcoholic EEG signals, such as inactivity of left hemisphere, slow wave of alcoholic EEG signals. Based on these conclusions, it can be deduced that the proposed HVGE method is very robust and efficient for alcoholic EEG signal analysis and classification. It will be also useful for other biomedical signal recognition.

## Electronic supplementary material

Below are the links to the electronic supplementary material.

(PS 16 kb)

(PS 16 kb)
